# (Poly)phenolic compounds and gut microbiome: new opportunities for personalized nutrition 

**DOI:** 10.20517/mrr.2022.06

**Published:** 2022-04-27

**Authors:** Luca Narduzzi, Vicente Agulló, Claudia Favari, Nicole Tosi, Cristiana Mignogna, Alan Crozier, Daniele Del Rio, Pedro Mena

**Affiliations:** ^1^Human Nutrition Unit, Department of Food & Drug, University of Parma, Parma 43125, Italy.; ^2^Phytochemistry and Healthy Foods Lab (LabFAS), Food Science and Technology Department (CEBAS-CSIC), University Campus of Espinardo, Murcia 30100, Spain.; ^3^Department of Chemistry, King Saud University, Riyadh 11451, Saudi Arabia.; ^4^School of Medicine, Dentistry and Nursing, University of Glasgow, Glasgow G12 8QQ, United Kingdom.; ^5^Microbiome Research Hub, University of Parma, Parco Area delle Scienze 11/A, Parma 43124, Italy.

**Keywords:** (Poly)phenol, cardiometabolic health, metabotype, gut microbiota, personalized nutrition

## Abstract

For decades, (poly)phenols have been linked to cardiometabolic health, but population heterogeneity limits their apparent efficacy and the development of tailored, practical protocols in dietary interventions. This heterogeneity is likely determined by the existence of different metabotypes, sub-populations of individuals metabolizing some classes of (poly)phenols differently. The gut microbiota plays a major role in this process. The impact of microbiota-related phenolic metabotypes on cardiometabolic health is becoming evident, although the picture is still incomplete, and data are absent for some classes of (poly)phenols. The lack of a complete understanding of the main microbial actors involved in the process complicates the picture. Elucidation of the mechanisms behind phenolic metabotypes requires novel experimental designs that can dissect the inter-individual variability. This paper, in addition to providing an overview on the current state-of-the-art, proposes wider metabotyping approaches as a means of paving the way towards effective personalized nutrition with dietary (poly)phenols.

## INTRODUCTION

Modern societies struggle with the economic and social consequences of the rise in cardiometabolic diseases (CMD), a cluster of metabolic dysfunctions (e.g., dyslipidemia, hypertension, insulin resistance, impaired glucose tolerance, etc.) that may lead to systemic impairments like cardiovascular diseases and type 2 diabetes^[1]^. Unhealthy dietary habits, including excessive consumption of free sugars, saturated fats, and sodium, and low intake of vegetables and fruits, among others, are recognized as key determinants of the onset of CMD, while the promotion of healthy diets represents a cornerstone for public health policies. Data suggest that more than 30% of all deaths could be prevented through dietary changes, particularly by increasing the consumption of plant-based foods^[2,3]^. Indeed, adequate nutrition has been established as the most important prevention factor in CMD^[4]^, as healthy dietary patterns supply all the necessary nutrients for the human body and promote health. These benefits have been related to the direct action of these nutrients and the proliferation of a healthy microbiota in the human intestine^[5]^. This is relevant as several indirect factors are associated with cardiometabolic risk, namely lipid metabolism including cholesterol transport, short chain fatty acids production, and vascular inflammation, which are known to be mediated by the gut microbiota. 

Extensive research is shedding light on the health benefits of plant-based diets and specific plant foods. Besides providing energy, fiber and essential micronutrients, plant-based foods are rich sources of a variety of phytochemicals with well-known health-promoting effects, the so-called plant bioactives^[6]^. Some bioactive phytochemicals such as (poly)phenols, carotenoids, phytosterols, glucosinolates, alkaloids, thiosulfinates, and alkylresorcinols may promote health and well-being by preventing obesity, type 2 diabetes, and cardiovascular diseases^[3,6-8]^. Among them, (poly)phenols are the most diverse category of plant bioactive compounds, widespread in fruits, vegetables, cereals, and beverages including fruit juices, wine, coffee, beer, and tea. They constitute a heterogeneous family, and are classified into flavonoids [flavonols, flavan-3-ols (both monomers and proanthocyanidins), flavanones, flavones, isoflavones, and anthocyanins, and non-flavonoids including phenolic acids, hydrolysable tannins, lignans, and stilbenes]. Growing evidence from clinical trials and cohort studies suggests that an increased intake of (poly)phenols may reduce the risk of CMD and obesity^[9-11]^. (Poly)phenol-rich diets reduce blood pressure and improve blood lipid profile, avoid endothelial dysfunction through a nitric-oxide mediated decrease of pro-inflammatory cytokines, inhibit platelet function and aggregation in blood vessels, prevent insulin resistance through pancreatic β-cells protection, control glycemic response lowering blood glucose and transport, and avoid low-grade inflammation, among other features^[12,13]^. However, these beneficial effects may vary among individuals due to differences in gut microbiota composition and functionality, among other factors, defining the singularity of each human being.

## (POLY)PHENOLS, GUT MICROBIOTA, AND CARDIOMETABOLIC DISEASES: A COMPLEX RELATIONSHIP

Despite the conspicuous body of evidence, the relationship between (poly)phenol intake and cardiometabolic prevention is enigmatic. A wide variability has been highlighted within and between studies, undermining the significance of the role of (poly)phenols in CMD prevention. Apart from the expected variability due to the administration procedure, the response to dietary (poly)phenols has been demonstrated to be heterogeneous among subjects and populations, leading to inconclusive results. Consequently, the role of (poly)phenols in cardiometabolic health promotion has not yet been demonstrated consistently^[14,15]^. It is clear that the studies attempting to link (poly)phenols with the prevention of CMD have not taken all the relevant variables into account. One or more pieces of the puzzle are still missing.

### (Poly)phenol bioavailability and metabolism: the role of gut microbiome

**Figure 1:**
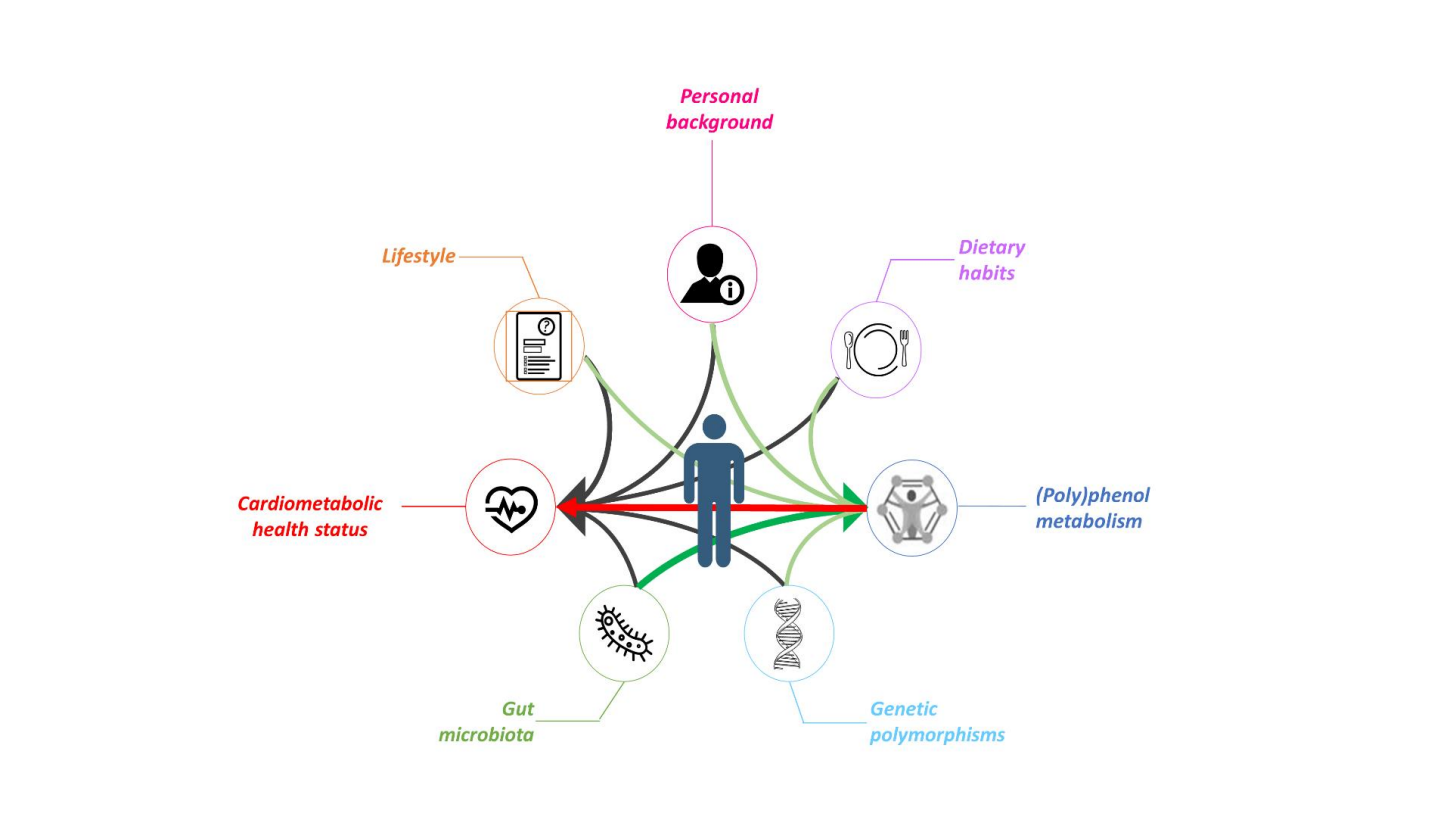
All the factors reported in this figure directly influence cardiometabolic health (black arrows). (Poly)phenol metabolism has an important role in cardiometabolic health (red arrow) and is influenced by other factors, especially the gut microbiota (green arrows).

The heterogeneity observed in response to (poly)phenol consumption can be associated primarily with inter-individual differences in their bioavailability^[16]^. These differences are attributed to several factors, including genetic background, gut microbiome, sex, age, medication, and lifestyle habits such as diet, smoking, and physical activity [Figure 1]. There is, however, a growing body of evidence indicating that the gut microbiome plays a key role in (poly)phenol metabolism and absorption and is believed to underpin much of the inter-individual variation observed in their bioavailability^[16]^.

Ingested (poly)phenols present in foods mainly as glycosides and esterified forms, and while they can be absorbed to some extent in the upper gastrointestinal tract, substantial amounts none-the-less arrive at the colon, where they encounter the resident microbiota. The microbiota can cleave conjugated moieties releasing aglycones and perform ring-fission, dehydroxylation, and other reactions, transforming the native (poly)phenolic compounds into a plethora of (low-molecular-weight) catabolites that can be absorbed by colonocytes and subjected to phase II metabolism by colonocytes and hepatocytes before entering the systemic circulation. Some phenolics originating from microbial catabolism exhibit higher bioactivity than their parent compounds. This is the case with dihydroresveratrol (resveratrol catabolite) and equol (daidzein catabolite)^[17]^. However, the array of gut microbial metabolites is highly variable between individuals, due to the diversity of bacteria species and strains comprising the flora of the colonic microbiota. 

The relationship between gut microbiota and (poly)phenols is bidirectional: gut microbiota is involved in the bioconversion of (poly)phenols, affecting their bioavailability, metabolism, and bioactivity, while phenolic compounds can positively modulate the colonic microbial composition and function. (Poly)phenols can affect the intestinal ecology and preserve the gut microbial balance exerting: (i) a prebiotic effect by enhancing the growth and establishment of probiotic bacterial families such as Bifidobacteriaceae and Lactobacillaceae, and (ii) an antimicrobial activity by inhibiting pathogenic bacteria such as *Escherichia coli*, *Clostridium perfrigens*, *Clostridium histolyticum*, and *Helicobacter pylori*^[18]^. The concept of the “three P’s for gut health”, comprising probiotics, prebiotics, and (poly)phenols, has been proposed, and the classical concept of “prebiotics”, restricted to certain dietary carbohydrates, has been re-evaluated^[19]^. 

A “prebiotic-like” effect has been reported for some phenolic compounds and some (poly)phenol-derived gut microbial metabolites using both *in vitro* assays with human gut microbiota and *in vivo* preclinical studies and clinical trials, with phenolic compounds and (poly)phenol-rich foods such as grape seed, wine, cocoa, cranberry, green tea, blueberry, nuts, and pomegranate^[19]^. (Poly)phenols can also modify the gut microbiota: (i) favoring specific gut microorganisms that may provide health benefits to the host, such as *Akkermansia muciniphila*, *Faecalibacterium prausnitzii*, *Enterococcus, Prevotella, Roseburia*, and *Bacteroides* spp.; and (ii) reducing the *Firmicutes/Bacteroidetes* ratio, which is higher in obese and metabolic syndrome subjects^[20]^. A recent study shows that (poly)phenol intake during early life in mice promotes *Akkermansia* growth and host colonic barrier improvement in association with an increase in *Lactobacillus*-secreted lactate, suggesting that (poly)phenol consumption may be beneficial also in early life^[21]^.

### Handling population-wise variability in (poly)phenol metabolism: metabotypes

Since (poly)phenols are extensively metabolized by the gut microbiota, inter-individual differences in bacteria-converting species can contribute to inter-person variations. This results in an extensive variability in the concentration of phenolic metabolites in circulation, as has been demonstrated for: (i) flavanones, with the production of several phenolic acids^[22]^; (ii) hop prenylflavonoids, with the production of 8-prenylnaringenin; (iii) lignans, with the production of enterolactone; and (iv) flavan-3-ols, with the production of phenyl-γ-valerolactones and phenyl-propanoic acids^[23-25]^. Phenotypical differences can also be related to the selective production of specific metabolites, like equol (soy isoflavone daidzein derivative), dihydro-avenanthramides (oat avenanthramides derivatives), or urolithins (ellagic acid/ellagitannin derivatives)^[26-28]^. 

**Figure 2:**
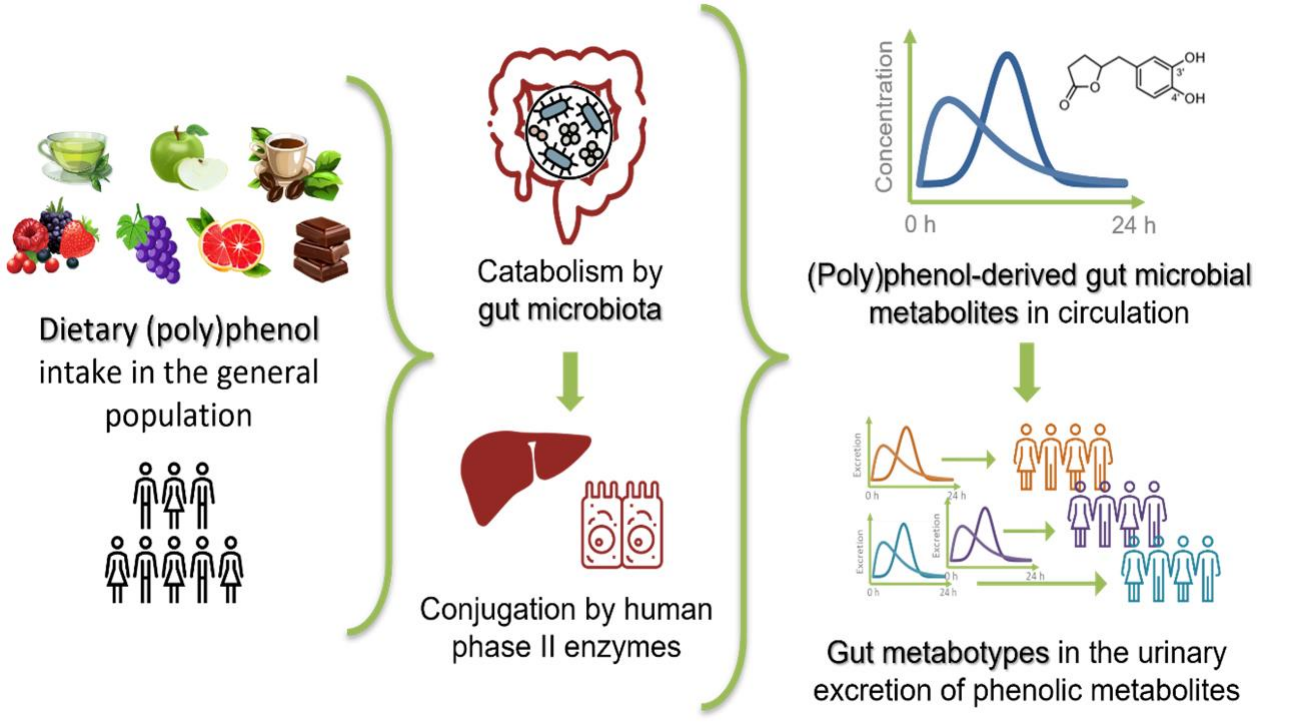
(Poly)phenol intake and metabolism in the general population and metabotype identification according to the excretion of gut microbial metabolites.

The different production patterns of gut microbiota-derived (poly)phenol metabolites can be related to the existence of metabolic phenotypes (namely “metabotypes”) in the population. According to earlier research^[19]^, the term “gut metabotype” refers to a metabolic phenotype defined by: (i) the existence of specific gut microbial metabolites characterizing the metabolism of the parent phenolic compound; and (ii) the associated gut microbiota in terms of composition and activity. This definition, based on a qualitative criterion (binary response of production versus non-production), is of interest and fits well some metabolites (e.g., equol, dihydro-avenanthramides, and urolithins). However, it may be restrictive when addressing the inter-individual variation in the metabolism of most phenolics, characterized by production gradients. A broader meaning, based on a quali-quantitative criterion, has been proposed by our research group when considering the metabolism of green tea and cranberry flavan-3-ols^[24,29]^. This approach for “phenolic metabotypes” is based on the metabotype concept commonly accepted withinside the nutrition field as “subgroups of individuals sharing the same metabolic profile”^[30]^. Undeniably, a wide concept of gut-mediated phenolic metabotypes may also assist in addressing the inter-individual variability in the production of (poly)phenol-derived gut microbial metabolites when the subjects produce all or most of the phenolic metabolites of a catabolic pathway, but in different proportions, as it happens for flavan-3-ols and other classes of dietary (poly)phenols^[23-25,29,31]^ [Figure 2].

The stratification of individuals according to their qualitative phenolic profile has been assessed for the metabolism of ellagitannins/ellagic acid and isoflavones. Subjects can be classified into three urolithin metabotypes (UM) according to the excretion of urolithins after consumption of ellagitannins or ellagic acid-rich foods, mainly walnuts and pomegranate, but also berries including raspberries and strawberries. Metabotype A (UM-A) is characterized by the excretion of urolithin-A (Uro-A) and its phase II conjugates. Metabotype B (UM-B) produces conjugates of isourolithin-A (IsoUro-A) and urolithin-B (Uro-B) in addition to urolithin-A while metabotype 0 (UM-0) does not produce Uro-A, IsoUro-A, or Uro-B derivatives^[32]^. UM-0 shows lower microbiome diversity and richness than UM-A and UM-B. A study has reported that 3-day walnut consumption already modulates the gut microbiota in a UM-dependent manner and boosts the production of short-chain fatty acids^[33]^. So far, two urolithin-producing species, *Gordonibacter urolithinfaciens* and *G. pamelaeae*, belonging to the Coriobacteriaceae family, have been isolated from human feces. The Coriobacteriia class is increased in UM-B versus UM-A and UM-0 and is positively associated with BMI and cardiometabolic risk factors^[34]^.

Regarding the metabolism of soy isoflavone daidzein, equol-producing and *O*-desmethylangolensin (ODMA)-producing metabotypes have been identified. These metabotypes are unrelated; the capacity to harbor equol-producing bacteria is not associated with the capacity to favor ODMA-producing bacteria. Caucasian and Asian populations display a distinct distribution of these metabotypes, and this difference cannot be shortened by regular consumption of high-isoflavone-containing products^[35]^. The equol metabotypes show a different abundance of equol-producing bacteria and a different microbial composition^[34]^. More than ten gut microbes are involved in equol production, including *Slackia equolifaciens*, *Slackia isoflavoniconvertens*, and *Adlercreutzia equolifaciens. *Reports regarding gut microbes able to produce ODMA are less abundant^[35]^. 

**Figure 3:**
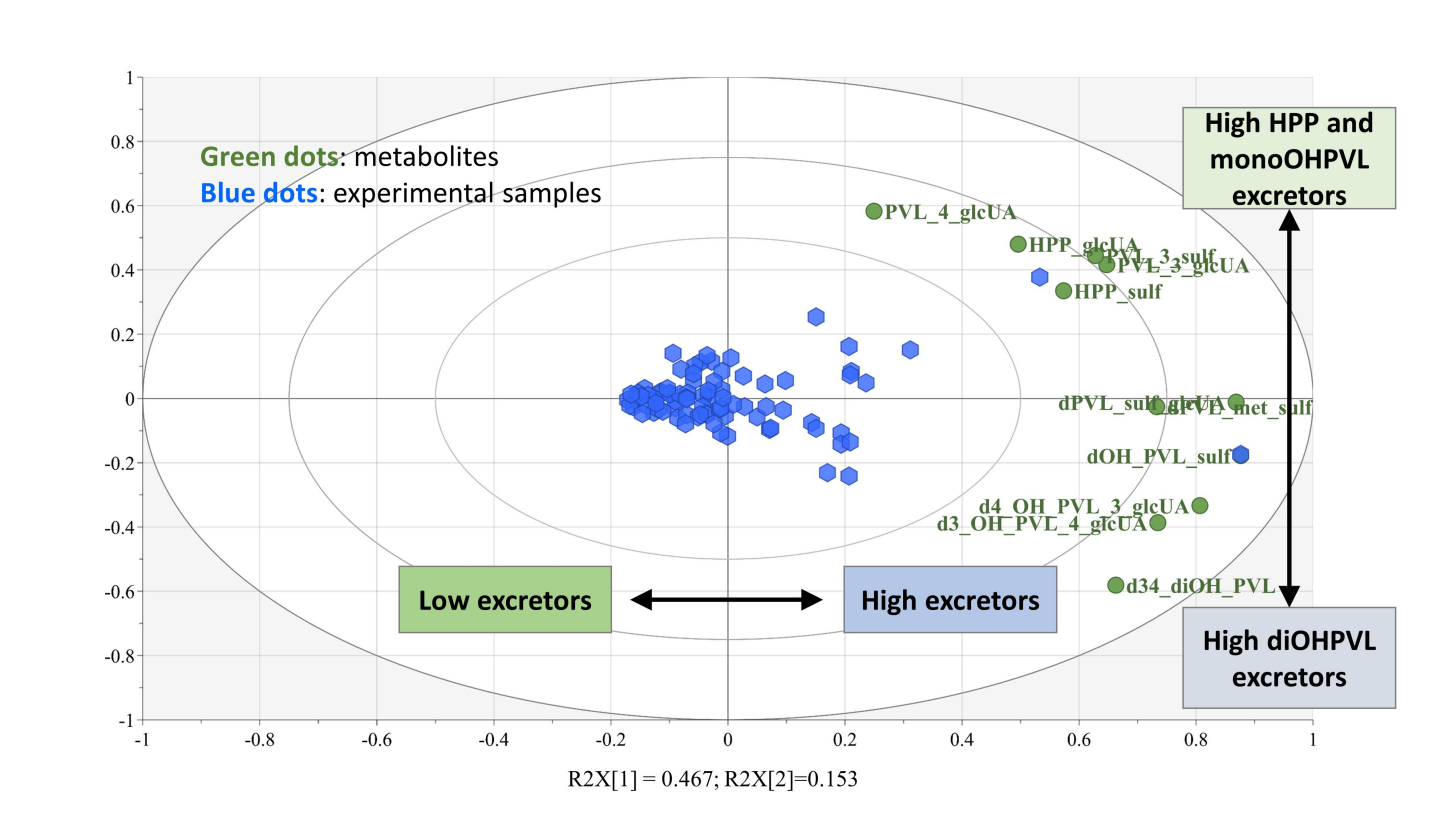
Different profiles in the urinary excretion of flavan-3-ols colonic metabolites after consumption of cranberry. PCA figure modified from Mena *et al.*(2021)^[29]^. Abbreviations: PVL_3_sulf: 5-phenyl-γ-valerolactone-3’-sulfate, PVL_3_glcUA: 5-phenyl-γ-valerolactone-3’-glucuronide; PVL_4_glcUA: 5-phenyl-γ-valerolactone-4’-glucuronide; d34_diOH_PVL: 5-(3’,4’-dihydroxyphenyl)-γ-valerolactone; dOH_PVL_sulf: (hydroxyphenyl)-γ-valerolactone-sulfate (3’,4’ isomers); d4_OH_PVL_3_glcUA: 5-(4’-hydroxyphenyl)-γ-valerolactone-3’-glucuronide; d3_OH_PVL_4_glcUA: 5-(3’-hydroxyphenyl)-γ-valerolactone-4’-glucuronide; dPVL_met_sulf: 5-phenyl-γ-valerolactone-methoxy-sulfate isomer (3’,4’); dPVL_sulf_gluc: 5-phenyl-γ-valerolactone-sulfate-glucuronide isomer (3’,4’); HPP_sulf: 3-(phenyl)propanoic acid-sulfate; HPP_glcUA: 3-(phenyl)propanoic acid-glucuronide, diOHPVL = 5-(3’,4’-dihydroxyphenyl)-γ-valerolactone derivatives; monoOHPVL: 5-(hydroxyphenyl)-γ-valerolactone derivatives, both 3’ and 4’ isomers; HPP: 3-(hydroxyphenyl)propanoic acid derivatives.

Metabotyping has also been attempted for flavan-3-ols, the main dietary (poly)phenols. However, the picture here requires a comprehensive metabotype concept that can handle the heterogeneity of structures characterizing this subclass of flavonoids and the complexity of their catabolic pathways. Putative metabotypes have been proposed *in vivo* based on a different quali-quantitative urinary production of phenyl-γ-valerolactones (PVLs) and 3-(hydroxyphenyl)propanoic acids (HPPs) after consumption of (epi)gallocatechin-rich green tea. Three metabotypes have been identified: metabotype 1 characterized by high excretion of tri- and di-hydroxyPVLs and reduced excretion of HPPs, metabotype 2 distinguished by a medium excretion of dihydroxyPVLs and a limited excretion of trihydroxyPVLs and HPPs, and metabotype 3 characterized by limited production of PVLs and high amounts of HPPs^[24]^. Further studies reported a similar picture, although the precursor flavan-3-ols were different from those in green tea; low and high producers of flavan-3-ols colonic metabolites have been observed in two different studies, one performed *in vitro* fermenting pure (−)-epicatechin and the second one *in vivo* administering cranberry products^[29,36]^. In the *in vitro* study, anaerobic incubations of (−)-epicatechin with fecal inocula from 24 healthy individuals revealed different catabolic patterns, with some individuals able to convert 5-(3′,4′-dihydroxyphenyl)-γ-valerolactone into 5-(hydroxyphenyl)-γ-valerolactones and HPPs at a faster rate compared to others^[36]^. This result was also reflected in the urinary excretion of flavan-3-ol colonic metabolites in different human studies with cranberry products^[29]^, as can be observed in the PCA presented in Figure 3. Nevertheless, despite the robustness of the evidence for colonic metabotypes of flavan-3-ols, further ad hoc confirmatory research is needed to consolidate this result and fully understand what the gut microbiota species lie behind these differences and which may be their health-related consequences. Unfortunately, information on specific bacterial strains responsible for the catabolism of flavan-3-ols into PVLs and low molecular weight phenolics is very scarce. *Lactobacillus plantarum* IFPL935, *Eggerthella lenta*, *Adlercreutzia equolifaciens*, and *Flavonifractor plautii* are currently the only bacteria identified as being able to convert flavan-3-ols into 5-(3′,4′-dihydroxyphenyl)-γ-valerolactone and 5-(3′-hydroxyphenyl)-γ-valerolactone^[37-39]^. Moreover, no microorganisms responsible for further conversion into hydroxyphenylvaleric acids and HPPs have yet been identified.

Metabotypes based on a quantitative criterion have been reported for the microbiota-derived metabolites of flavanones, hop prenylflavonoids and lignans. Indeed, it has been observed that among the human population, three different metabotypes arise, and they can be classified in three quantitative metabotypes: poor, moderate and strong producers of 8-prenylnaringenin (for isoxanthohumol) and enterolactone and enterodiol (for lignans)^[22,23,40]^. Some studies have explored the possibility of inducing a switch in the metabotypes by increasing the intake of specific (poly)phenols. Long-term intake of isoflavones increases the level of equol production in equol producers, likely due to a metabolic adaptation of the gut microbiota. Nonetheless, boosting the ability to produce equol in non-equol producers has shown to be impractical. A study found that long-term intake of a high dose of isoflavones did not result in such a change^[41]^. In contrast, another, albeit low-powered study, reported that three among five non-equol producer women acquired the ability to produce equol after two weeks of soymilk consumption^[42]^. An explanation for these contrasting results might be that the microbial species involved in equol production differ among individuals, while the absence of equol-producing bacteria in the colon impairs the capability to produce equol. The percentage of equol producers has been estimated to be around 30% and 50%-60% in the Caucasian and Asian populations, respectively, suggesting that equol production is linked to ethnic background, which covers genetic background, gut microbiota composition, and dietary habits, including long-term isoflavone consumption^[34]^. 

Regarding urolithin metabotypes, evidence suggests that some individuals included in UM-0 can be converted to UM-A or UM-B producers after a long-term/high-dose intake of ellagitannins and ellagic acid, the so-called “UM-0 responders”^[43]^. Moreover, the distribution of UM-A and UM-B is critically affected by aging, while the proportions of individuals within UM-0 (about 10% of the total) remain constant from 5 to 90 years of age. At an early age, the proportion of UM-A accounts for about 85% of the population, progressively decreasing to 55% from 40 to 90 years of age, concomitant with an increment of UM-B from 15% up to 45%. The conversion from UM-A to UM-B happens mostly between 25 to 35 years of age; thereafter, the proportion of UM-A and UM-B (55% and 45%, respectively) remains more or less constant^[34]^.

### Impact of metabotypes on cardiometabolic health

To date, the possible effects of (poly)phenols on individual human health have not been fully identified. These could be due to: (i) the metabolites deriving from gut microbiota catabolism and/or phase-II conjugation; and/or (ii) the gut microbial ecology associated with (poly)phenol metabolism, among other factors^[34]^. Moreover, recent evidence indicates that the gut microbiota, independently and/or interactively with dietary intake, is a target for reducing cardiovascular disease risk through its effects on cardiometabolic risk factors. Dietary (poly)phenols may be a source for these interactive effects, and clustering of subjects into known metabotypes (i.e., metabotyping) could be a strategy to explain, at least in part, the different responses of individuals to (poly)phenol intake. 

There is evidence of the relationship between cardiovascular risk and phenotypes in the metabolization of ellagitannins and isoflavones, paving the way towards using metabotypes as cardiometabolic risk biomarkers and biomarkers of the potential (poly)phenol health effects mediated through gut microbiota^[19]^. In the case of ellagitannins, UM-A seems to be a protective metabotype, while UM-B seems to be a dysbiotic-prone metabotype associated with cardiometabolic impairments. The UM explains individual differences not only in baseline cardiovascular risk but also in the cardioprotective effect upon pomegranate consumption^[43]^. In particular, a study described an improvement in cardiometabolic biomarkers after pomegranate consumption only in overweight-obese subjects belonging to UM-B, suggesting a differential response to ellagitannin/ellagic acid intake according to the metabolic phenotype^[42]^.

Regarding isoflavones, an increasing number of studies associate daidzein-related metabotypes with cardiovascular risk, suggesting that equol and/or ODMA producers may have a lower risk than non-producers^[19,44]^. Acute vascular benefits have been reported after administering synthetic equol to men with an equol-producer metabotype, suggesting that the microbial ecology associated with the equol-producing capacity is crucial for determining health benefits^[45]^. In contrast, another study described a significant improvement of cardiometabolic risk biomarkers after chronic daily oral intake of *S*-equol, particularly in equol non-producer females^[46]^. Overall, it is unclear whether the presence of specific microbial communities associated with equol and/or ODMA production are involved in the effects exerted by these gut metabolites. Therefore, more intervention studies that stratify individuals according to their metabotype are necessary to explain the effects exerted by dietary isoflavones and assess if these protective effects are produced by the metabolites associated with (or without) the gut microbiota.

## THE NEED OF METABOTYPING TO PURSUE PERSONALIZED NUTRITION

Recommendations promoted at a population level in a “one-size-fits-all approach” for plant-based food consumption do not ensure that everyone is adequately exposed to and benefits from the protective features of plant bioactives. Evidence indicates that personalized nutritional recommendations may result in improved dietary behaviors. In this context, metabotyping (grouping individuals with comparable metabolic/phenotypic profiles) could pave the way forward in terms of personalized and targeted nutrition, improving health at a population level^[47]^. Using metabotypes to identify subgroups responding differentially to dietary interventions and to examine associations with cardiometabolic risk factors is turning into reality. The use of metabotypes in longitudinal studies demonstrates that they can be associated with cardiometabolic risk factors, whereas application in clinical studies serves to identify metabotypes with differential responses^[47]^.

Metabotyping may also be a key success factor in personalized approaches when considering (poly)phenols, providing tailor-made nutritional recommendations to lower disease risks. This concept mirrors the idea of precision medicine already developed to treat human diseases. A conspicuous corpus of literature indicates that there exists a wide inter-individual variability influencing the response of patients to treatments (including drugs) and prevention strategies, mostly due to genetic and environmental factors. Therefore, there is a need to develop a precision medicine able to take into account this variability and suggest a subject-specific approach to improve the benefits and reduce the harm of the treatments^[48]^. Similarly, individuals’ stratification through their (poly)phenol-producer metabotypes is necessary for intervention trials as specific metabotypes may produce the metabolites responsible for differential health effects. (Poly)phenol-metabolizing phenotypes can reflect different gut microbiota composition and metabolic status and could be biomarkers of health effects of (poly)phenols grounded on differential responses. Nonetheless, the potential application of metabotyping to evaluate the effect of (poly)phenols on cardiometabolic health, while considering factors of inter-individual variability, some points need to be addressed to guide the provision of effective strategies for the prevention of CMD:

1. Approaches focused on individual classes of (poly)phenols and their related phenolic metabolites are reductionist. Although it is important to attain fundamental knowledge and develop the field of study, these restricted approaches are hardly translated into effective nutritional guidelines. 

2. Metabotyping studies are both challenging and time-consuming as they require screening of participants and targeted recruitment for a particular phenotypic characteristic. Moreover, they must guarantee that they are adequately powered to evaluate the effect of dietary (poly)phenols both within and between specific population groups. 

3. Although metabotyping is key for targeted nutrition of population subgroups, sound tailored dietary advice with (poly)phenol-containing foods requires a more holistic view, including individual characteristics (precision nutrition).

4. The potential of metabotypes to predict cardiometabolic risk factors is currently promising. Beyond classical group analysis (metabotype x response), machine-learning algorithms can integrate multiple individual datasets to develop robust prediction models for the response to a dietary intervention.

5. Studies on the metabotype-wise mechanisms of action are completely lacking. In this sense, a must-do step would be to deploy omics studies to correlate the (poly)phenol effects on CMD to specific markers of effect in each metabotype. This would help the comprehension of the biochemical basis underlying personalized nutrition.

Overcoming these five limitations with appropriate study designs and analytical approaches would lead to conceptual and methodological progress. In this sense, the development and application of novel approaches may drive the success of strategies addressed to improve cardiometabolic health at an individual level based on the differential response to dietary (poly)phenols. Future knowledge of phenolic metabotypes will favor a better understanding of the gut microbiome and its interactions with host health, leading to new opportunities for personalized nutrition in the framework of plant-based diets. Furthermore, a comprehensive understanding of the role of specific gut-derived metabolites and gut microorganisms can lead to the development of preventive/therapeutic strategies that, according to the principles of precision medicine, could be applied in tailored interventions to increment or reduce the bioavailability of such metabolites or drive gut microbiota composition in specific populations, aiming to improve their cardiometabolic health.

## DECLARATIONS

### Copyright

© The Author(s) 2022.
